# A Prospective Study of Nephrocalcinosis in Very Preterm Infants: Incidence, Risk Factors and Vitamin D Intake in the First Month

**DOI:** 10.3390/medicina60121910

**Published:** 2024-11-21

**Authors:** Rasa Garunkstiene, Ruta Levuliene, Andrius Cekuolis, Rimante Cerkauskiene, Nijole Drazdiene, Arunas Liubsys

**Affiliations:** 1 Neonatology Centre, Vilnius University Hospital Santaros Klinikos, LT-08406 Vilnius, Lithuania; 2 Neonatal Department, Obstetrics and Gynecology Clinic, Vilnius City Clinical Hospital, LT-10207 Vilnius, Lithuania; 3 Institute of Applied Mathematics, Vilnius University, LT-03225 Vilnius, Lithuania; ruta.levuliene@mif.vu.lt; 4 Clinic of Children’s Diseases, Institute of Clinical Medicine, Vilnius University Faculty of Medicine, LT-03101 Vilnius, Lithuania; 5 Paediatrics Centre, Vilnius University Hospital Santaros Klinikos, LT-08406 Vilnius, Lithuania

**Keywords:** nephrocalcinosis, preterm infants, vitamin D

## Abstract

*Background and objectives*: Nephrocalcinosis (NC) is a common condition characterized by the deposition of calcium salts in the kidneys of very preterm infants due to tubular immaturity, intensive treatment and nutritional supplements. However, optimal vitamin D supplementation remains unclear. In most patients, NC spontaneously resolves within the first year of life, but long-term kidney function data are lacking. The aim was to study nephrocalcinosis in very preterm infants, assess risk factors and evaluate vitamin D’s impact during the first month with a 2-year follow-up. *Material and Methods*: This was a prospective observational study conducted over a 3-year period in infants with a gestational age of less than 32 weeks. The patients’ data were compared between the NC and control groups based on kidney ultrasound results at discharge. In the first month, the mean vitamin D intake from all sources as well as biochemical markers of calcium metabolism were collected. Patients diagnosed with NC were referred to a pediatric nephrologist after discharge. *Results*: NC was found in 35% of a cohort of 160 infants, more common in those with a gestational age <28 weeks. Risk factors were associated with higher morbidity and necessary treatment. At 28 days, serum 25-hydroxy vitamin D levels differed between NC and control groups (*p* < 0.05). The NC group with GA ≥ 28 weeks had higher vitamin D intake (*p* < 0.05), hypercalciuria and calcium/creatinine ratio (*p* < 0.01) and lower parathyroid hormone levels (*p* < 0.05). Follow-up showed resolution in 70% at 12 months and 90% at 24 months. *Conclusions*: The prevalence of NC in very preterm infants is significant, associated with lower maturity and higher morbidity. Careful vitamin D supplementation and biochemical monitoring of Ca metabolism from the first month of life should support bone health and limit the risk of nephrocalcinosis. Due to the high incidence of NC in very preterm infants, long-term follow-up is essential.

## 1. Introduction

Nephrocalcinosis (NC) is a common condition in very preterm infants, and it has been reported in 6–41% of infants [1,2,3,4,5,6]. The etiology of NC is multifactorial and comprehensive [2]. Various causes and contributors predispose neonates to developing NC, including metabolic, genetic (mutations in renal tubular transporters, channels and receptors) and iatrogenic factors [5]. NC in preterm infants involves the deposition of calcium salts in the kidneys due to tubular immaturity, complex treatment in the neonatal intensive care unit and supplements that promote calcium excretion or deposition [2]. Vitamin D supplementation can cause hypercalcemia, hypercalciuria and NC [6,7,8,9]. The current recommendation varies from 200–400 IU/day as per the American Academy of Pediatrics [10] to 400–700 IU/kg/d for stable preterm infants by the European Society for Pediatric Gastroenterology Hepatology and Nutrition (ESPGHAN) [11]. But the optimal dose and duration of vitamin D supplementation to improve bone density for very preterm infants and its association with NC are unclear [7,12]. Recommendations suggest an optimal concentration range of serum 25-hydroxy vitamin D (25(OH)D) of 30–50 ng/mL (75–125 nmol/L) [13]. Further, other metabolic factors are also involved [14]. Parathyroid hormone (PTH) activates vitamin D synthesis in the immature kidneys. Insufficient active vitamin D can cause an imbalance of calcium (Ca) and phosphorus (P) levels in the bloodstream, pushing PTH beyond its typical range [2,15,16]. Therefore, to maintain Ca metabolism balance, the regular monitoring of PTH, Ca and P levels, along with kidney function, is essential to manage and prevent complications in very preterm infants.

In the majority of patients, the spontaneous resolution of NC occurs in the first year of life [2,5,17]. However, NC may lead to calcium nephrolithiasis in particular conditions [5,17,18]. Nephrocalcinosis in preterm infants is associated with reduced kidney growth and volume, shorter length in the first year of life and potential long-term sequelae for glomerular and tubular function [19,20]. Furthermore, prematurity itself is associated with high blood pressure, relatively small kidneys and (distal) tubular dysfunction. Therefore, the long-term follow-up of blood pressure and renal glomerular and tubular function in preterm infants, especially those with neonatal nephrocalcinosis, seems warranted [20].

This prospective study aimed to assess the occurrence and risk factors of nephrocalcinosis in very preterm infants born at less than 32 weeks of gestation. Additionally, the impact of vitamin D intake in the first month was evaluated. Follow-up was conducted over a period of 2 years.

## 2. Materials and Methods

### 2.1. Study Population and Design

This was a prospective observational study carried out in the tertiary-care Neonatology Center at the Vilnius University Hospital Santaros Klinikos. Infants born at <32 weeks’ GA during a 3-year period were enrolled after parental informed consent. Infants were excluded due to death before 28 days of age, major congenital defects or no kidney ultrasound results. This study was approved by the Vilnius Regional Biomedical Research Ethics Committee and was registered in the Clinical Trials Registry (ClinicalTrials.gov Identifier: NCT04382976).

### 2.2. Data Collection

Patients’ data were collected and compared between NC and control groups as well as by GA (<28 and ≥28 weeks) based on kidney ultrasound results at discharge. Data collected included demographic, clinical, biochemical and treatment results during the hospital stay from the medical records. General information about maternal characteristics was inserted.

The baseline levels of pH, sodium, ionized Ca, serum Ca and serum 25-hydroxy vitamin D (25(OH)D) were collected in the first week before vitamin D supplementation. At the 28-day mark, we collected the same biochemical blood samples, along with the additional analysis of phosphorus, creatinine, iPTH (biologically active form of PTH) and alkaline phosphatase (ALT) levels, as well as urine spot samples, following the hospital protocol for metabolic bone disease (MBD) in preterm infants. Therefore, this process did not involve any additional blood test. A diagnosis of MBD was made based on a urine tubular reabsorption of phosphate rate greater than 95%.

Kidney ultrasound was performed using a linear probe (GE 11L-D) on a GE LogiqS8 ultrasound unit by a single pediatric ultrasound specialist (A.C.). Kidney microcalcifications were documented as absent or present. If present, they were evaluated as unilateral or bilateral, solitary or multiple—scattered or in groups. Attention was paid to the presence and character of corticomedullar differentiation (normal, inverse, absent).

All patients identified to have NC were referred to the pediatric nephrologist (R.C.) for follow-up after discharge.

### 2.3. Nutrition Protocol and Vitamin D Intake

Vitamin D intake was precisely counted in micrograms (5 µg—200 IU) as the average of the day from parenteral and enteral sources during the first 28 days. The delivery of vitamin D was ensured via parenteral nutrition containing vitamin D2 from fat-soluble vitamin supplements (Vitalipid N, Fresenius Kabi Uppsala, Sweden), 160 IU (4 µg)/kg/daily from the second day of life. The infants were given expressed mother’s or donor’s milk, which was fortified with Aptamil ProExpert (Nutricia) once the enteral intake reached 80–100 mL/kg/day. None of the infants were on formula feeds. The vitamin D content of a mothers’ own milk was approximately 2 IU/100 mL, and the added milk fortifier contained approximately 202 IU/100 mL [12]. Moreover, from day 8, the infants received oral supplementation with 500 IU (12.5 µg) of vitamin D3 every second day using Aquadetrim drops (15,000 IU/mL, Medana Pharma SA, Sieradz, Poland). The management of vitamin D and milk fortification, as well as all clinical decisions (due to the toleration of milk fortification, the duration of parenteral nutrition, etc.), were at the discretion of the attending physician on the clinical service.

### 2.4. Statistical Analysis

A comparative analysis of the study and control groups was performed. Descriptive statistics of the characteristics were obtained. For quantitative variables, the means and standard deviations were computed. In the case of qualitative variables, frequencies and percentages were presented. The Shapiro–Wilk test was performed to check the assumption of the normality. As the data were not normally distributed, the Wilcoxon rank-sum test was used. Logistic regression was used to adjust for potential confounders associated with NC. The results of the multivariate analysis were expressed in odds ratios (ORs) with a 95% confidence interval (95% CI). In the case of nominal variables, groups were compared using the Fisher exact test. Hypotheses were tested using a significance level of 0.05. In the case of quantitative variables, the tables show the mean ± standard deviation (mean ± s. d.); qualitative characteristics are presented by frequencies and percentages. Data entry was conducted in Microsoft Excel spreadsheets. The data obtained were analyzed by using the IBM SPSS Statistics 29.0 program (IBM Corp., Armonk, NY, USA).

## 3. Results

**Figure 1:**
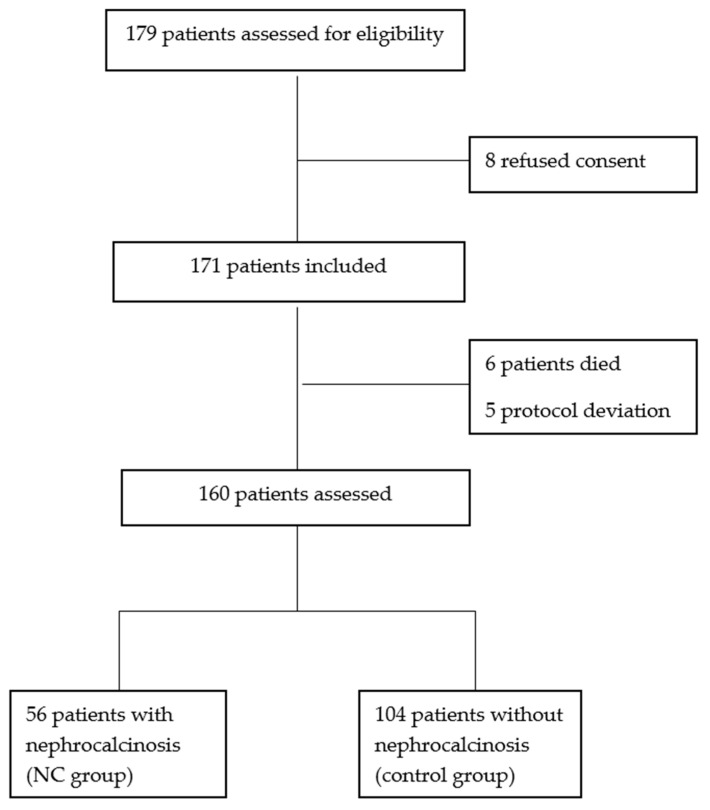
Flow chart.

Over the course of a three-year period, 179 eligible preterm infants were admitted (Figure 1). Eight parents refused consent, and eleven failed to complete the study. Therefore, 160 infants participated in the study, with 56 (35%) in NC group. Sixty-nine infants were less than 28 weeks of GA. NC was more common in those with a GA < 28 weeks (33 of 69 neonates; 47.8%, *p* < 0.05). In the ≥28 weeks group, 23 of 91 had NC (25.3%, *p* > 0.05).

The general characteristics are provided in Table 1. The mothers’ characteristics of these two groups were similar. Family history of nephrolithiasis was not comparable because of a lot of missing data. NC infants were significantly less mature and had lower GAs and smaller birth weights. They had lower Apgar scores; longer hospital stays; and more persistent ductus arteriosus, surfactant use and blood transfusions than non-NC infants. Moreover, the frequency and duration of invasive ventilation were longer in the NC group. When comparing infants based on GA, a significantly higher number of infants with a GA ≥ 28 weeks and NC required ventilation. However, there was no difference in ventilation rates among infants with a GA < 28 weeks.

**Figure 2:**
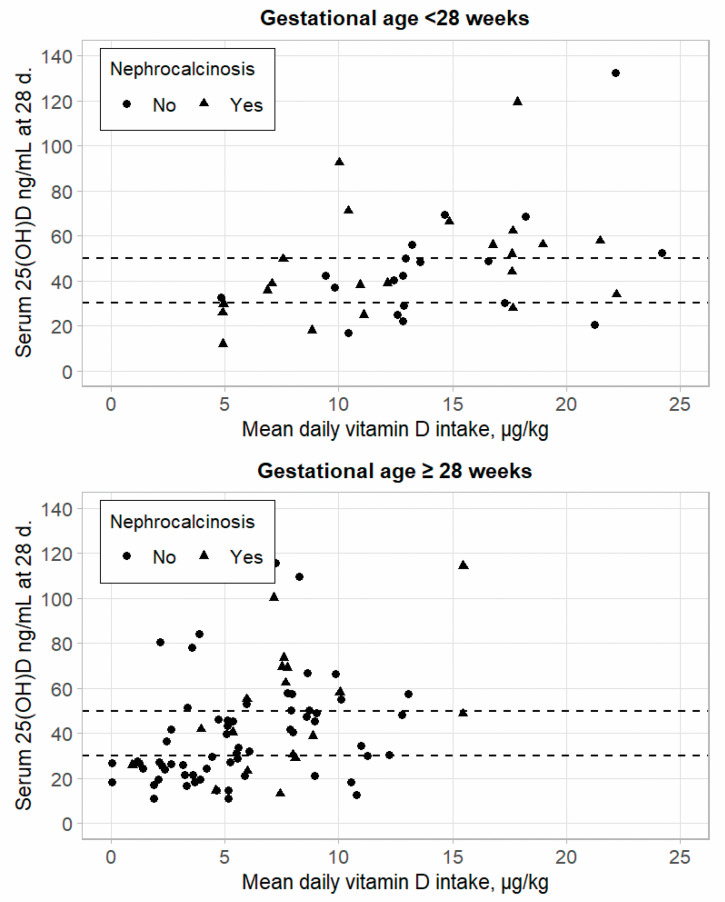
Average vitamin D intake in µg/kg during the first 28 days (5 µg—200 IU). Serum 25(OH)D—serum 25-hydroxyvitamin D; d.—days. Different symbols for control ● and nephrocalcinosis ▲ groups. Serum 25(OH)D levels at 30 and 50 ng/mL (optimal concentration) are marked as dotted lines.

The mean daily vitamin D intake of infants from all sources during the first 28 days was compared between groups and by GA using the Wilcoxon test (Figure 2). In the intake, we saw a wide dissemination, especially in the group with GA < 28 weeks. However, in the group with GA ≥ 28 weeks and NC, the mean daily vitamin D intake was significantly higher than in the control group (8.14 µg/kg = 325.6 IU/kg vs. 5.66 µg/kg = 226.4 IU/kg; *p* < 0.05). Moreover, in the group with GA < 28 weeks, the mean daily vitamin D intake had no statistical difference between NC and the control groups (12.83 µg/kg = 513.2 IU/kg vs. 14.32 µg/kg = 572.8 IU/kg). These intakes were within ESPGAN recommendations [11].

**Figure 3:**
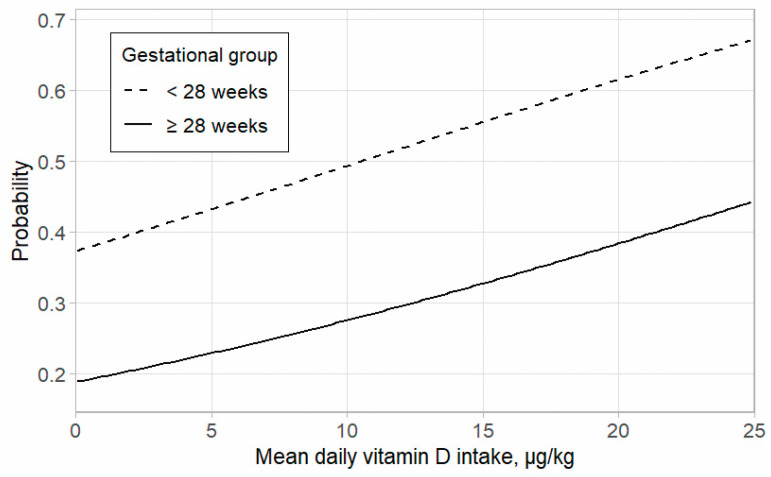
Probability of nephrocalcinosis by gestational age and the mean daily intake of vitamin D. Gestational groups (<28 weeks and ≥28 weeks of gestational age) are presented with different line types.

Figure 3 shows a plot of the effect of the regressors on the modeled probability of having NC. We could see that the probability of NC increased with increasing vitamin D intake, but not significantly. The probability of NC was higher in the lower GA group.

**Figure 4:**
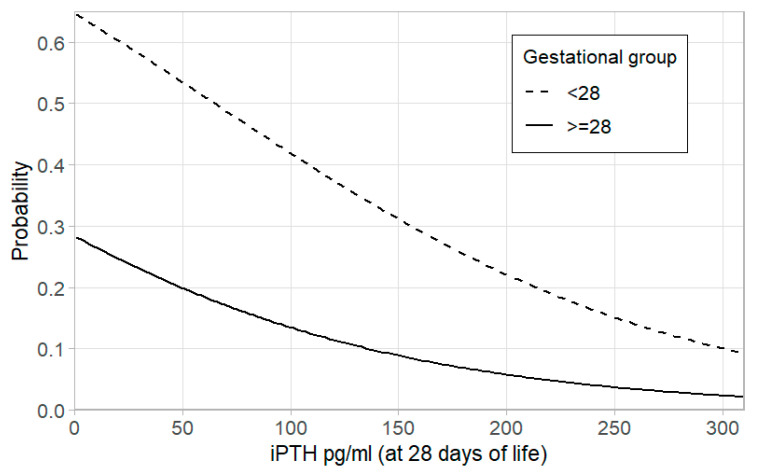
A decreasing trend in the probability of nephrocalcinosis for gestational age is observed when parathyroid hormone (at 28 days of life) increases. iPTH—intact parathyroid hormone. Gestational groups (<28 weeks and ≥28 weeks of gestational age) are presented with different line types.

Table 2 shows the results of laboratory tests compared by GA during the first 28 days of life. No differences were observed in the first blood analysis tests. The baseline level of serum 25(OH)D concentration in the first week before vitamin D supplementation showed vitamin D deficiency (<20 ng/mL) in 54% of patients, with no significant difference among the groups and gestation. On the 28th day of life, the serum 25(OH)D concentration was significantly higher in the NC group (49.68 ± 28.72 ng/mL (124.2 ± 71.8 nmol/L)) than in the control group (39.92 ± 22.88 ng/mL (99.8 ± 57.2 nmol/L)) (*p* < 0.05); however, there was no difference by GA (Table 2). When comparing the blood levels of factors involved in Ca metabolism across different GAs (iPTH at GA ≥ 28 weeks and ALT at GA < 28 weeks), they were lower in the NC group than in the control group (*p* < 0.05). In addition, a decreasing trend in the probability of NC was observed when iPTH (at 28 days of life) increased (OR (based on 10 units change) 0.911, 95% CI 0.834–0.994, *p* = 0.08) (Figure 4). Furthermore, the group of infants with GA ≥ 28 weeks was less likely to have NC (OR 0.216, 95% CI 0.110–0.425, *p* = 0.0002) when we compared the iPTH levels to infants with GA < 28 weeks. Moreover, in the group with GA ≥ 28 weeks and NC, there was significantly higher hypercalciuria levels and Ca/creatinine ratios than in the control group (*p* < 0.01). Significant differences in laboratory test results and probability trends related to factors influencing NC are observed in very preterm infants at 28 days of life.

The NC group was followed for two years to monitor the dynamic of NC. Out of the 56 infants with NC, follow-up was conducted for 45 of them. Of 45 infants with NC, 30 (66.7%) had persistent NC at 6 months of corrected age (KA), 13 (28.8%) at 12 months of KA, and 2 (4.4%) at 24 months of KA. All infants had normal glomerular filtration rates.

## 4. Discussion

In the current study, NC was observed in 35% of infants born at less than 32 weeks’ gestation, which is consistent with previously reported studies. Our data indicate that the likelihood of NC is higher, with a rate occurring twice as frequently in infants born before 28 weeks’ GA (47.8%) compared to infants born at 28 to 32 weeks’ GA (25.3%) [5,8]. Gestational age and lower birth weight were the significant risk factors for NC, which aligns with the results of many studies [2,5,6,8]. This is attributed to the immature function of kidneys during the neonatal period, impacting optimal Ca excretion. Another potential possibility is an association between severe morbidity and occurrence of NC, as we observed [1,5,15,17]. These infants were sicker, requiring intensive care and prolonged hospitalization. The multivariate analysis conducted by Chang et al. [1] revealed that comorbidities independently contribute to the risk of NC. Various studies provide divergent findings regarding sex ratio, morbidity and treatment [1,2,5,8,17]. Fayad et al.’s study [8] disclosed comparable median durations of respiratory support between the cases and control groups. We found a longer duration in the NC group, with a higher number of patients in the ≥28 weeks of GA group. Respiratory support in preterm infants can involve additional diuretic treatment which impacts the development of NC [3,8]. However, in our case, this was not a significant factor because diuretics were not used routinely.

Whereas NC can be a manifestation of an underlying genetic disorder (mutations in renal tubular transporters, channels, and receptors), neonates with NC must undergo an evaluation to identify and address contributors and to prevent further renal calcium deposition that can potentially lead to renal disfunction [5]. However, no genetic testing was performed in our study. The patients identified with NC were followed up by a pediatric nephrologist after discharge.

The probability of developing NC increases as the average vitamin D dosage rises. In our study, it was observed that the mean intake of vitamin D over 28 days from all sources was statistically significantly higher in the NC group with GA ≥ 28 weeks. In the group with GA < 28 weeks, our data on vitamin D intake showed considerable variability, with no statistically significant difference between the groups, but with a high prevalence of NC. In the study by Mauras et al. [3], it was stated that there is significantly less dispersion with the adapted supplementation protocol, which includes adjusting the vitamin D dose. Additionally, this protocol likely reduces the risk of both rickets and NC in this population. In a retrospective study, Fayad et al. [8] found that vitamin D intake was similar in both NC and control groups. However, nutritional support must be well monitored to support postnatal growth and limit the risk of nephrocalcinosis. It is challenging to manage the exact dose for very preterm infants in the group with GA < 28 weeks. The pathways of vitamin D absorption and metabolism are fully operative in infants born at <28 weeks’ GA. Based on this, recommendations for daily vitamin D intake in stable preterm infants have been established [10,11]. Many studies on preterm infants have compared responses to different levels of vitamin D supplementation with these guidelines in mind [7,12,21,22,23,24]. However, in these studies, the incidence of NC was not analyzed or was only partially observed in the group with increased vitamin D levels [22].

In our group of preterm infants with GA <32 weeks, 54% had baseline serum 25(OH)D deficiency. Vitamin D deficiency at birth varied by region and gestational age. For example, in a study with infants born at <28 weeks’ GA, vitamin D deficiency was observed in 67% of cases [21], whereas in infants born at <33 weeks’ GA, it was observed in 30% of cases [22]. At 28 days of life, our study found that NC was associated with higher serum 25(OH)D concentration, regardless of GA. This concentration falls within the recommendations for Central Europe, where the optimal range is 30–50 ng/mL (75–125 nmol/L) [13]. In our study, we did not detect hypercalcemia in any infants in the first month of life; however, hypercalciuria was significant in the NC ≥28 weeks GA group, which is consistent with other data [8]. Vitamin D supplementation is essential for optimal bone formation, but studies speculate that higher target should probably not exceed 48 ng/mL (120 nmol/L) [9]. In our population, we did not find any difference in MBD number based on the biochemical analysis. However, we found that in the NC group, according to GA, there was significantly less PTH (at ≥28 weeks) and ALP (at <28 weeks). As it is known, PTH tends to be sensitive as an early indicator of MBD [13,16]. According to available data, the relationships between vitamin D supplementation, serum 25(OH)D concentration, parathyroid hormone level, hypercalciuria and development of NC in preterm infants are not well established [6,25]. When vitamin D activation is impaired, PTH compensates by attempting to regulate Ca and P levels, sometimes leading to deviations from the normal range [2,16,25]. Recent research has explored the relationship between PTH and serum 25(OH)D during the postnatal period. Remarkably, an inverse correlation exists between these two factors. The study conducted by Taylor et al. investigated the association between serum 25(OH)D status and markers of bone health in very preterm infants. Interestingly, this association follows a linear pattern until a threshold of 44 ng/mL (110 nmol/L) for 25(OH)D status, beyond which, PTH plateaus [24]. In our study, we observed that in the NC group, the concentration of 25(OH)D exceeded the previously mentioned threshold associated with good bone mineralization. Additionally, among infants with a GA ≥ 28 weeks’, data showed homogeneity along with clear evidence of hypercalciuria, higher daily vitamin D intake and lower PTH level in the first month of life. Noteworthily, an inverse trend of probability of nephrocalcinosis in this group was observed when PTH levels increased at 28 days of life. So, understanding the complex factors related to NC in preterm infants is essential for future monitoring and clinical management.

Although the spontaneous resolution of NC occurs in most cases, data on long-term kidney function in preterm infants who presented with NC during this critical development period are lacking [8]. Nephrocalcinosis in preterm neonates can impact volume and the growth of kidneys and can cause long-term sequelae for kidney function [19,20,26]. Therefore, it is recommended to monitor both kidney growth and function based on ultrasonographic findings and general clinical health. In our population, NC resolved in around 70% at 12 and 90% at 24 months of KA. Our infants had normal kidney function at follow-up. NC-related monitoring includes kidney ultrasonography, serum creatinine measurement, and blood pressure measurement [2,5,8]. Additionally, the urine calcium-to-creatinine ratio may be indicated to detect and monitor hypercalciuria. Therefore, it is recommended to monitor both kidney growth and function.

This study had several limitations. First, this was a relatively small, single-center study. Second, decisions concerning the management of vitamin D and milk fortification were at the discretion of the attending physician and were not completely standardized. Therefore, there are no practical recommendations regarding the supplementation of vitamin D in relation to the development of NC in preterm infants. Third, no genetic testing was performed in this study, which would have allowed for an individual approach to the patient. Furthermore, follow-up was not completed for all infants.

## 5. Conclusions

The prevalence of NC in very preterm infants (<32 weeks GA) is significant and associated with lower maturity and higher morbidity. Careful vitamin D supplementation and biochemical monitoring of calcium metabolism, including PTH, from the first month of life should support bone health and limit the risk of nephrocalcinosis. The follow-up of kidney function in these infants is needed.

## Figures and Tables

**Table 1:** General characteristics.

Maternal Characteristics		Nephrocalcinosis (56)	Control Group (104)	*p*-Value
Age at delivery (years)		31.27 ± 4.32	31.54 ± 4.29	NS
Multiple pregnancy, n (%)		24 (42.86%)	36 (34.62%)	NS
Antenatal steroids, n (%)		47 (83.93%)	94 (90.38%)	NS
Hypertension, n (%)		11 (19.64%)	25 (24.04%)	NS
Mode of delivery (s/c), n (%)		30 (53.57%)	65 (62.5%)	NS
Infants’ characteristics				
Gestational age, weeks		27.59 ± 1.99	28.34 ± 2.13	*p* < 0.05
Gestational age, n (%)	<28 weeks	33 (58.93%)	36 (34.62%)	*p* < 0.05
	≥28 weeks	23 (41.07%)	68 (65.38%)	NS
Birth weight, grams		1093.0 ± 351.8	1185.8 ± 338.4	*p* < 0.05
Apgar score at 1 min		6.59 ± 1.80	7.25 ± 1.62	*p* < 0.01
Apgar score at 5 min		7.70 ± 1.33	8.22 ± 1.23	*p* < 0.001
Sex, n (%)	Male	38 (67.86%)	59 (56.73%)	NS
	Female	18 (32.14%)	45 (43.27%)	NS
Hospitalization NICU, days		18.85 ± 2	15.89 ± 14.91	*p* < 0.01
Hospitalization total, days		74.73 ± 23.37	64.47 ± 23.22	*p* < 0.01
Age at discharge, weeks		38.14 ± 2.24	37.45 ± 1.91	NS
Intraventricular hemorrhage I°, n (%)		7 (12.5%)	18 (17.31%)	NS
Intraventricular hemorrhage II°, n (%)		11 (19.64%)	12 (11.54%)	NS
Persistent ductus arteriosus (PDA), n (%)		24 (42.86%)	27 (26.21%)	*p* < 0.05
Sepsis, n (%)		9 (16.07%)	16 (15.38%)	NS
Metabolic bone disease, n(%)		27 (48.21%)	43 (41.35%)	NS
Treatment				
Antibiotics, n (%)		56 (100%)	102 (98.08%)	NS
Dexametason, n (%)		6 (10.71%)	5 (4.81%)	NS
Surfactant, n (%)		45 (80.36%)	65 (63.11%)	*p* < 0.05
Vasopressors, n (%)		12 (21.43%)	19 (18.27%)	NS
Blood transfusion, n (%)		21 (37.5%)	20 (19.23%)	*p* < 0.05
PDA, medications, n (%)		14 (25%)	17 (16.35%)	NS
PDA, operation, n (%)		4 (7.14%)	6 (5.77%)	NS
Invasive ventilation, days		7.21 ± 11.00	4.32 ± 8.70	*p* < 0.05
Invasive ventilation, n (%)		32 (57.14%)	39 (37.5%)	*p* < 0.05
Invasive ventilation, n (%)	<28 weeks	22 (66.67%)	22 (61.11%)	NS
	≥28 weeks	10 (43.48%)	17 (25%)	*p* < 0.05
Noninvasive ventilation, n (%)		55 (98.21%)	103 (99.04%)	NS
Caffeine, weeks		34.96 ± 2.20	34.89 ± 2.65	NS
Parenteral nutrition, days		8.95 ± 3.62	9.33 ± 6.75	NS

The data are expressed as mean (±standard deviation) or median (IQR). n—number; NICU—neonatal intensive care unit; PDA—persistent ductus arteriosus; NS—not significant (*p* > 0.05).

**Table 2:** Results of laboratory tests (compared by gestational age).

Characteristic	Gestational Age < 28 Weeks			Gestational Age ≥ 28 Weeks		
	Nephrocalcinosis	Control Group	*p*-Value	Nephrocalcinosis	Control Group	*p*-Value
First week						
Blood serum analysis						
pH	7.34 ± 0.05	7.32 ± 0.06	NS	7.15 ± 1.06	7.36 ± 0.05	NS
iCa mg/dL	5.2 ± 0.44	5.12 ± 0.36	NS	4.8 ± 0.88	5.12 ± 0.36	NS
Sodium mg/dL	558.4 ± 31.2	549.2 ± 24.32	NS	545.6 ± 24.16	551.2 ± 17.8	NS
25(OH)D ng/mL	23.31 ± 11.51	18.98 ± 12.28	NS	20.91 ± 10.91	21.14 ± 9.8	NS
Common Ca mg/dL	9.36 ± 0.76	9.36 ± 0.84	NS	9.44 ± 0.64	12.24 ± 21.76	NS
Day 28						
Blood serum analysis						
pH	7.35 ± 0.07	7.35 ± 0.07	NS	7.39 ± 0.05	7.36 ± 0.05	NS
iCa mg/dL	5.32 ± 0.24	5.32 ± 0.2	NS	5.4 ± 0.16	5.4 ± 0.24	NS
Sodium mg/dL	530 ± 19.6	523.6 ± 24.84	NS	535.2 ± 10.84	537.2 ± 11.48	NS
25(OH)D ng/mL	47 ± 23.88	41.43 ± 23.8	NS	53.37 ± 34.29	39.41 ± 22.7	NS
Common Ca mg/dL	9.92 ± 0.44	9.84 ± 0.52	NS	10.32 ± 0.36	10 ± 0.64	NS
Phosphorus mg/dL	8.56 ± 0.92	8.36 ± 1.64	NS	9.04 ± 0.72	8.88 ± 0.92	NS
Creatinine ng/mL	0.57 ± 0.26	0.58 ± 0.14	NS	0.50 ± 0.12	0.49 ± 0.07	NS
iPTH pg/mL	48.98 ± 51.91	69.37 ± 69.75	NS	22.76 ± 20.45	35.51 ± 25.98	*p* < 0.05
ALP U/L	570.0 ± 202.5	698.6 ± 236.4	*p* < 0.05	455.4 ± 148.3	470.6 ± 133.4	NS
Urine analysis						
Calcium mg/dL	11.08 ± 6.96	12.28 ± 8.72	NS	18.36 ± 10.12	12.68 ± 11.4	*p* < 0.01
Phosphorus mg/dL	22.8 ± 20.24	36.48 ± 41.28	NS	12.64 ± 17.64	8.88 ± 13.56	NS
Creatinine mg/dL	4.16 ± 1.72	4.88 ± 2.96	NS	4.36 ± 3.6	4.04 ± 2.76	NS
Ca/creatinine ratio mg/mg	0.96 ± 0.5	0.88 ± 0.37	NS	1.87 ± 1.06	1.18 ± 0.78	*p* < 0.01

Values are presented as mean ± standard deviation. iCa—ionized calcium; 25(OH)D—serum 25-hydroxyvitamin D; Ca—calcium; iPTH—intact parathyroid hormone (biologically active form of PTH); ALP—alkaline phosphatase; NS—not significant (*p* > 0.05).

## Data Availability

The data presented in the current study are available on reasonable request from the corresponding author.
